# Establishment and Validation of an Integrated Model to Predict Postoperative Recurrence in Patients With Atypical Meningioma

**DOI:** 10.3389/fonc.2021.754937

**Published:** 2021-10-07

**Authors:** Xiao-Yong Chen, Jin-Yuan Chen, Yin-Xing Huang, Jia-Heng Xu, Wei-Wei Sun, Yue- Chen, Chen-Yu Ding, Shuo-Bin Wang, Xi-Yue Wu, De-Zhi Kang, Hong-Hai You, Yuan-Xiang Lin

**Affiliations:** ^1^ Department of Neurosurgery, Neurosurgical Research Institute, The First Affiliated Hospital, Fujian Medical University, Fuzhou, China; ^2^ Department of Ophthalmology, The First Affiliated Hospital, Fujian Medical University, Fuzhou, China; ^3^ Department of Neurosurgery, Fuzong Clinical Medical College of Fujian Medical University, Fuzhou, China; ^4^ Fujian Key Laboratory of Precision Medicine for Cancer, The First Affiliated Hospital, Fujian Medical University, Fuzhou, China; ^5^ Key Laboratory of Radiation Biology of Fujian Higher Education Institutions, The First Affiliated Hospital, Fujian Medical University, Fuzhou, China

**Keywords:** atypical meningioma, recurrence, predict, LASSO, nomogram, model

## Abstract

**Background:**

This study aims to establish an integrated model based on clinical, laboratory, radiological, and pathological factors to predict the postoperative recurrence of atypical meningioma (AM).

**Materials and Methods:**

A retrospective study of 183 patients with AM was conducted. Patients were randomly divided into a training cohort (*n* = 128) and an external validation cohort (*n* = 55). Univariable and multivariable Cox regression analyses, the least absolute shrinkage and selection operator (LASSO) regression analysis, time-dependent receiver operating characteristic (ROC) curve analysis, and evaluation of clinical usage were used to select variables for the final nomogram model.

**Results:**

After multivariable Cox analysis, serum fibrinogen >2.95 g/L (hazard ratio (HR), 2.43; 95% confidence interval (CI), 1.05–5.63; *p* = 0.039), tumor located in skull base (HR, 6.59; 95% CI, 2.46-17.68; *p* < 0.001), Simpson grades III–IV (HR, 2.73; 95% CI, 1.01–7.34; *p* = 0.047), tumor diameter >4.91 cm (HR, 7.10; 95% CI, 2.52–19.95; *p* < 0.001), and mitotic level ≥4/high power field (HR, 2.80; 95% CI, 1.16–6.74; *p* = 0.021) were independently associated with AM recurrence. Mitotic level was excluded after LASSO analysis, and it did not improve the predictive performance and clinical usage of the model. Therefore, the other four factors were integrated into the nomogram model, which showed good discrimination abilities in training cohort (C-index, 0.822; 95% CI, 0.759–0.885) and validation cohort (C-index, 0.817; 95% CI, 0.716–0.918) and good match between the predicted and observed probability of recurrence-free survival.

**Conclusion:**

Our study established an integrated model to predict the postoperative recurrence of AM.

## Introduction

Meningioma is a common primary brain tumor that comprises about 36.4% of all central nervous system (1). According to the 2016 World Health Organization (WHO) grading criterion (2), meningioma has been classified into three grades. WHO grade II meningioma, which is named as atypical meningioma (AM), is rare and more progressive and invasive compared with WHO grade I meningioma, with 5-year recurrence rates ranging from 30% to 60% after surgical resection (3–6). Therefore, identifying predictive factors for recurrence is important to individually manage AM patients. To date, reliable prediction for recurrence of AM patients remains challenging.

At present, pathological diagnosis is the gold standard for the diagnosis of AM. The incidence of this disease is relatively small, and it accounts a relatively small proportion in meningioma. Therefore, prospective studies for AM are difficult to perform. Performing additional retrospective reviews on AM patients could analyze and summarize the characteristics and risk factors for recurrence of those patients, which could provide assistance for preoperative AM diagnosis, postoperative recurrence prediction, and personalized follow-up regimen development. Many factors, including age, extent of resection, tumor location, mitotic index, Ki-67 index, postoperative radiation therapy (PORT), and serum biomarkers have been identified as effective predictive factors of recurrence and prognosis in AM (7–11). However, the current evidence exploring the risk factors for recurrence in AM patients remains equivocal. Considering the limited precision and effectiveness of a single risk factor, an integrated model with multiple factors may be more suitable for recurrence prediction.

Here, we propose an integrated model based on clinical, laboratory, radiological, and pathological factors to predict the recurrence of AM patients after surgical resection, which assists us to predict the therapeutic effects in the heterogeneous patients and make individualized follow-up management.

## Method

### Study Cohort

The medical records of 183 patients diagnosed as AM who received surgical resection at the First Affiliated Hospital of Fujian Medical University and Fuzong Clinical Medical College of Fujian Medical University between January 2011 and June 2019 were retrospectively reviewed. The study was approved by the local ethics committee of the First Affiliated Hospital of Fujian Medical University and Fuzong Clinical Medical College of Fujian Medical University. It was conducted in accordance with the ethical guidelines of the Declaration of Helsinki. Informed consent was waived for this retrospective study. The eligibility criteria for inclusion were as follows: (1) age ≥18 years old; (2) diagnosis of AM was confirmed by pathological examination. Patients operated on before 2016 were examined for pathological results to confirm the diagnosis based on 2016 WHO criterion (2); (3) complete medical records including clinical, laboratory, imaging, and pathological information; (4) no history of surgical treatment and adjuvant therapy before admission; and (5) no other tumor, autoimmune, and inflammatory diseases. There were 29 AM patients which were excluded because of lack of specific information.

### Data Collection and Follow-Up

Patient information were retrieved from medical records at the first Affiliated Hospital of Fujian Medical University. For each patient, the following information was obtained: age, sex, comorbid condition, preoperative routine serum test, tumor features (location, size, peritumoral edema) based on computed tomography (CT) or magnetic resonance imaging (MRI), extent of resection (Simpson grades I–II or Simpson grades III–IV), skull invasion, immunohistochemical features (mitotic level and Ki-67 index), and PORT. Tumor features in the MRI were independently assessed by two experienced neurosurgeons who were blind to patient characteristics. Similarly, the pathological diagnoses of all patients were confirmed by two experienced pathologists according to the 2016 WHO CNS tumor grading criterion (2). Based on the preoperative routine serum test, neutrophil-to-lymphocyte ratio (NLR), platelet-to-lymphocyte ratio (PLR), lymphocyte–monocyte ratio (LMR), and systemic inflammatory response index (SIRI) were calculated as follows: NLR = neutrophil/lymphocyte, PLR = platelet/lymphocyte, LMR = lymphocyte/monocyte, and SIRI = monocyte × neutrophil/lymphocyte.

In addition to the first reexamination within 1 month after surgery, patients were regularly screened for recurrence by contrast-enhanced CT or MRI every 3 months in the first year, every 6 months in the second year, and annually thereafter. The primary endpoint was recurrence-free survival (RFS), defined as the time from surgery to initial recurrence (12).

The cutoff values of several serum biomarkers and tumor diameter for predicting tumor recurrence was determined by the receiver operating characteristic (ROC) curve analysis as follows, NLR = 2.59, PLR = 74.9, LMR = 5.46, SIRI = 0.77, serum fibrinogen (FIB) = 2.95 g/L, and tumor diameter = 4.91 cm.

### Model Building and Statistics

Considering the sample size of our study, we used training cohort and validation cohort without test cohort during modeling process. Patients were randomly assigned into training cohort (*n* = 128) and validation cohort (*n* = 55) at a common ratio of 7:3 (13) to avoid the potential bias associated with small sample size of validation set. Based on our own experience and previous studies, we hypothesized that a constellation of clinical, laboratory, imaging, and immunohistochemical parameters were related to the recurrence of AM. Continuous factors such as age, mitotic level, and Ki-67 index were turned into dichotomies as suggestions proposed by previous study (14). Univariable Cox regression analysis was initially utilized to identify potential predictive factors for tumor recurrence. Factors with a *p*-value less than 0.10 in the univariable Cox regression analysis were further analyzed by multivariable analysis. Nonsignificant factors (*p* ≥ 0.05) were removed from the model by forward elimination procedure. The factors left after the stepwise procedure of multivariable analysis were further included in the least absolute shrinkage and selection operator (LASSO) regression analysis in order to avoid overfitting or underfitting of the model. The models were compared before (model A) and after (model B) LASSO regression analysis based on predictive performance and clinical usage. Time-dependent ROC curve was utilized to evaluate the accuracy and effectiveness of the two models at different time points. Decision curve analyses (DCA), integrated discrimination improvements (IDI), and Net Reclassification Index (NRI) were applied to assess and compare the clinical usage of the two models.

After a comprehensive comparison, the final model was applied to establish a nomogram to predict the probability of RFS at 2, 3, and 5 years. In both training and validation cohorts, the discrimination ability of the nomogram was evaluated by Harrell’s concordance index (C-index), and the consistency between the actual and predicted RFS rate was confirmed by the calibration curve.

Statistical analysis was performed by SPSS 19.0 statistical software (SPSS, Inc., Chicago, IL, USA) and R statistical software (R version 4.0.3, R Project, www.r-project.org). Continuous variables were presented as mean ± standard deviation (data with normal distribution) for two-sample *t*-test or median (range) (data without normal distribution) for Mann-Whitney *U* test. Categorical variables were presented as frequency (percentage) and compared with Pearson Chi-square test or Fisher exact test. All statistical tests were two sided, and statistical significance was set at *p* < 0.05.

## Results

### Patient Characteristics

The general characteristics of all patients and comparison of two cohorts were presented in **Table 1**. Of the 183 patients, 63 patients were male and 120 patients were female; the proportion of patients with age ≥60 years was 33.9%. The proportion of recurrence (*p* = 0.154) showed gratifying similarity between the training cohort and the validation cohort. In addition, the other parameters showed the two cohorts were homogeneous and comparable, indicating that the data were reliable with high quality.

**Table 1:** Characteristics of patients in the training and validation cohorts.

Characteristic	All (*n* = 183)	Training cohort (*n* = 128)	Validation cohort (*n* = 55)	*p*-Value
**Demographics**				
Age				
<60 years	121 (66.1%)	80 (62.5%)	41 (74.5%)	0.114
≥60 years	62 (33.9%)	48 (37.5%)	14 (25.5%)	
Sex				
Male	63 (34.4%)	49 (38.3%)	14 (25.5%)	0.094
Female	120 (65.6%)	79 (61.7%)	42 (74.5%)	
**Comorbid condition**				
Hypertension				
No	151 (82.5%)	104 (81.3%)	47 (85.5%)	0.492
Yes	32 (17.5%)	24 (18.8%)	8 (14.5%)	
Diabetes mellitus				
No	175 (95.6%)	121 (94.5%)	54 (98.2%)	0.476
Yes	8 (4.4%)	7 (5.5%)	1 (1.8%)	
**Laboratory data**				
RBC count 10^9^/L	4.51 (4.17–4.80)	4.56 (4.17–4.87)	4.41 (4.18–4.71)	0.107
WBC count 10^9^/L	6.01 (5.10–7.40)	6.03 (5.09–7.46)	5.81 (5.10–7.40)	0.754
NEU count 10^9^/L	3.67 (2.92–5.19)	3.52 (2.83–5.11)	4.05 (3.16–5.35)	0.117
MON count 10^9^/L	0.35 (0.25–0.45)	0.35 (0.27–0.46)	0.35 (0.23–0.45)	0.535
LYM count 10^9^/L	1.82 (1.52–2.19)	1.82 (1.53–2.18)	1.76 (1.48–2.20)	0.757
PLT count 10^9^/L	236.00 (190.05–279.00)	234.50 (187.76–276.75)	236.25 (196.00–289.80)	0.431
NLR	2.00 (1.53–3.04)	1.95 (1.52–2.74)	2.61 (1.55–3.31)	0.073
PLR	125.22 (102.12–158.95)	121.22 (99.61–155.24)	131.25 (104.60–171.14)	0.296
LMR	5.58 (4.24–6.90)	5.32 (4.14–6.58)	5.91 (4.48–7.25)	0.209
SIRI	0.66 (0.42–1.10)	0.66 (0.42–1.10)	0.66 (0.42–1.10)	0.944
FIB (g/L)	2.75 (2.43–3.36)	2.69 (2.39–3.26)	2.81 (2.54–3.42)	0.165
HB (g/L)	132.26 ± 14.80	133.33 ± 15.69	129.76 ± 12.27	0.134
**Tumor features and surgical factors**				
Location				
Nonskull base	141 (77.0%)	101 (78.9%)	40 (72.7%)	0.362
Skull base	42 (23.0%)	27 (21.1%)	15 (27.3%)	
Tumor diameter (cm)	4.93 ± 1.39	4.91 ± 1.49	4.95 ± 1.11	0.859
Peritumoral edema				
≤1 cm	73 (39.9%)	52 (40.6%)	21 (38.2%)	0.757
>1 cm	110 (60.1%)	76 (59.4%)	34 (61.8%)	
Extent of resection				
Simpson grades I–II	158 (86.3%)	112 (87.5%)	46 (83.6%)	0.485
Simpson grades III–IV	25 (13.7%)	16 (12.5%)	9 (16.4%)	
Skull invasion				
No	118 (64.5%)	79 (61.7%)	39 (70.9%)	0.234
Yes	65 (35.5%)	49 (38.3%)	16 (29.1%)	
**Immunohistochemical feature**				
Mitotic level				
<4/HPF	116 (63.4%)	84 (65.6%)	32 (58.2%)	0.338
≥4/HPF	67 (36.6%)	44 (34.4%)	23 (41.8%)	
Ki-67 index				
<5%	113 (61.7%)	79 (61.7%)	34 (61.8%)	0.990
≥5%	70 (38.3%)	49 (38.3%)	21 (38.2%)	
**PORT**				
No	151 (82.5%)	109 (85.2%)	42 (76.4%)	0.151
Yes	32 (17.5%)	19 (14.8%)	13 (23.6%)	
**Recurrence**				
No	139 (76.0%)	101 (78.9%)	38 (69.1%)	0.154
Yes	44 (24.0%)	27 (21.1%)	17 (30.9%)	

Values are reported as number, number (%), median (25%–75%), and mean ± standard deviation.

RBC, red blood cell; HCT, hematocrit; WBC, white blood cell; NEU, neutrophil; MON, monocyte; LYM, lymphocyte; PLT, platelet; NLR, neutrophil-lymphocyte ratio; PLR, platelet-to-lymphocyte ratio; LMR, lymphocyte–monocyte ratio; SIRI, systemic inflammatory response index; FIB, fibrinogen; HB, hemoglobin; HPF, high-power field; PORT, postoperative radiation therapy.

The median length of follow-up, RFS, and overall survival of the included patients were 50.00 months (34.00–77.00), 25.50 months (14.00–37.25), and 34.00 months (31.00–48.00), respectively. In the 25 patients with Simpson grades III–IV, the patients with PORT had a longer RFS than those without PORT (54.00 *vs* 17.50 months, *p* = 0.034).

### Predictive Factors of Recurrence in the Training Cohort

The ROC curve analysis showed that NLR = 2.59, PLR = 74.90, LMR = 5.46, SIRI = 0.77, FIB = 2.95 g/L, and tumor diameter = 4.91 cm were the optimal cutoff values (**Table 2**). Based on the corresponding cutoff values, the area under curve (AUC) of NLR, PLR, LMR, SIRI, FIB, and tumor diameter were 0.638 (95% confidence interval (CI), 0.549–0.721), 0.503 (95% CI, 0.414–0.593), 0.550 (95% CI, 0.459–0.638), 0.570 (95% CI, 0.479–0.657), 0.679 (95% CI, 0.591–0.759), and 0.702 (95% CI, 0.615–0.780), respectively; the sensitivity of NLR, PLR, LMR, SIRI, FIB, and tumor diameter were 59.26%, 100.00%, 59.26%, 59.26%, 66.67%, and 74.07%, respectively; the specificity of NLR, PLR, LMR, SIRI, FIB, and tumor diameter were 75.25%, 7.92%, 55.45%, 65.35%, 75.25%, and 59.41%, respectively; the *Youden* index of NLR, PLR, LMR, SIRI, FIB, and tumor diameter were 0.345, 0.079, 0.147, 0.246, 0.419, and 0.335, respectively.

**Table 2:** The cutoff value and area under the curve of the possible predictive factors of recurrence in training cohort.

Parameter	Cutoff value	AUC	Sensitivity (%)	Specificity (%)	Youden index	95% CI of AUC	*p*-Value
NLR	2.59	0.638	59.26	75.25	0.345	0.549–0.721	0.026
PLR	74.90	0.503	100.00	7.92	0.079	0.414–0.593	0.957
LMR	5.46	0.550	59.26	55.45	0.147	0.459–0.638	0.433
SIRI	0.77	0.570	59.26	65.35	0.246	0.479–0.657	0.258
FIB	2.95	0.679	66.67	75.25	0.419	0.591–0.759	0.004
Tumor diameter cm	4.91	0.702	74.07	59.41	0.335	0.615–0.780	<0.001

AUC, area under curve; CI, confidence interval; NLR, neutrophil-to-lymphocyte ratio; PLR, platelet-to-lymphocyte ratio; LMR, lymphocyte–monocyte ratio; SIRI, systemic inflammatory response index; FIB, fibrinogen.

The univariable analysis showed that neutrophil count (HR, 1.13; 95% CI, 0.98–1.30; *p* = 0.092), NLR >2.59 (HR, 3.62; 95% CI, 1.67–7.82; *p* = 0.001), SIRI >0.77 (HR, 2.50; 95% CI, 1.14–5.47; *p* = 0.022), FIB >2.95 g/L (HR, 3.56; 95% CI, 1.65–7.69; *p* = 0.001), tumor located in skull base (HR, 2.63; 95% CI, 1.18–5.86; *p* = 0.018), Simpson grades III–IV (HR, 2.53; 95% CI, 1.07–5.98; *p* = 0.035), tumor diameter >4.91 cm (HR, 3.97; 95% CI, 1.68–9.41; *p* = 0.002), mitotic level ≥4/high-power field (HR, 2.21; 95% CI, 1.04–4.70; *p* = 0.040), and PORT (HR, 2.18; 95% CI, 0.95–5.00; *p* = 0.065) were associated with AM recurrence (**Table 3**). In the multivariable analysis, FIB >2.95 g/L (HR, 2.43; 95% CI, 1.05–5.63; *p* = 0.039), tumor located in skull base (HR, 6.59; 95% CI, 2.46–17.68; *p* < 0.001), Simpson grades III–IV (HR, 2.73; 95% CI, 1.01–7.34; *p* = 0.047), tumor diameter >4.91 cm (HR, 7.10; 95% CI, 2.52–19.95; *p* < 0.001), and mitotic level ≥4/high-power field (HR, 2.80; 95% CI, 1.16–6.74; *p* = 0.021) were independently associated with AM recurrence (**Table 3**).

**Table 3:** Univariable and multivariable cox hazard regression analyses of recurrence in the training cohort.

Parameter	Univariable analysis			Multivariable analysis		
	HR	95% CI	*p*-Value	HR	95% CI	*p*-Value
Age ≥60 years	1.72	0.81–3.65	0.162			
Male	0.60	0.28–1.28	0.184			
Hypertension	1.10	0.42–2.92	0.842			
Diabetes mellitus	0.05	0–92.41	0.427			
RBC	1.21	0.60–2.44	0.592			
WBC	1.11	0.96–1.27	0.167			
NEU	1.13	0.98–1.30	0.092			
MON	0.30	0.03–2.98	0.302			
LYM	0.93	0.45–1.90	0.833			
PLT	1.00	1.00–1.01	0.760			
NLR >2.59	3.62	1.67–7.82	0.001			
PLR >74.90	22.50	0.04–14518.00	0.346			
LMR >5.46	1.79	0.83–3.86	0.138			
SIRI >0.77	2.50	1.14–5.47	0.022			
FIB >.95 g/L	3.56	1.65–7.69	0.001	2.43	1.05–5.63	0.039
HB	1.00	0.98–1.03	0.825			
Tumor located in skull base	2.63	1.18–5.86	0.018	6.59	2.46–17.68	<0.001
Tumor diameter >4.91 cm	3.97	1.68–9.41	0.002	7.10	2.52–19.95	<0.001
Peritumoral edema > 1 cm	0.82	0.38–1.79	0.623			
Simpson grades III–IV	2.53	1.07–5.98	0.035	2.73	1.01–7.34	0.047
Skull invasion	1.47	0.69–3.13	0.321			
Mitotic level ≥4/HPF	2.21	1.04–4.70	0.040	2.80	1.16–6.74	0.021
Ki-67 index ≥5%	1.23	0.57–2.66	0.596			
PORT	2.18	0.95–5.00	0.065			

HR, hazard ratio; CI, confidence interval; RBC, red blood cell; WBC, white blood cell; NEU, neutrophil; MON, monocyte; LYM, lymphocyte; PLT, platelet; NLR, neutrophil-lymphocyte ratio; PLR, platelet-to-lymphocyte ratio; LMR, lymphocyte–monocyte ratio; SIRI, systemic inflammatory response index; FIB, fibrinogen; HB, hemoglobin; HPF, high-power field; PORT, postoperative radiation therapy.

### Variable Selection for Final Model Based on LASSO Regression Analysis and Time-Dependent ROC, DCA, IDI, and NRI

**Figure 1:**
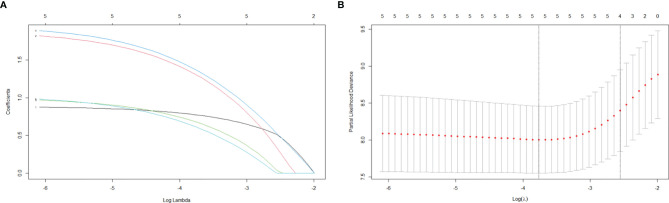
LASSO regression analysis for variable selection. **(A)** LASSO regression coefficients. **(B)** LASSO cross-validation.

The LASSO regression analysis was utilized to identify whether there were overfitting or underfitting in the five independent risk factors for the recurrence (**Figure 1**). The optimal *λ* (one standard error of the minimum criteria) was selected with a value of 0.08 and four nonzero coefficients. Considering the clinical importance of mitotic level and its exclusion by LASSO regression analysis, we established two models: ModelA, all the five independent risk factors including mitotic level; ModelB, all the independent prognostic factors without mitotic level (**Table 4**).

**Table 4:** The composition of two models based on lasso regression analysis.

	Model A			Model B		
	HR	95% CI	*p*-Value	HR	95% CI	*p*-Value
FIB >2.95 g/L	2.43	1.05–5.63	0.039	2.73	1.20–6.19	0.016
Tumor located in skull base	6.59	2.46–17.68	<0.001	4.42	1.87–10.45	0.001
Simpson grades III–IV	2.73	1.01–7.34	0.047	2.77	1.06–7.22	0.038
Tumor diameter >4.91 cm	7.10	2.52–19.95	<0.001	4.94	1.99–12.25	0.001
Mitotic level ≥4/HPF	2.80	1.16–6.74	0.021			

HR, hazard ratio; CI, confidence interval; FIB, fibrinogen; HPF, high-power field.

**Figure 2:**
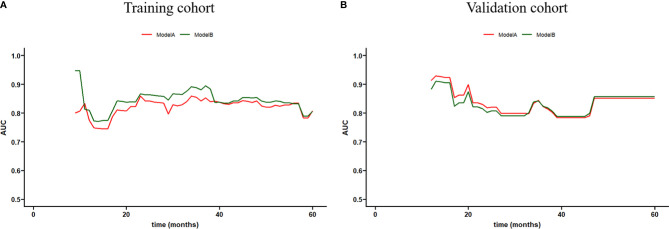
Time-dependent receiver operating characteristic (ROC) curve of models **(A, B)** in the training and validation cohorts.

**Figure 3:**
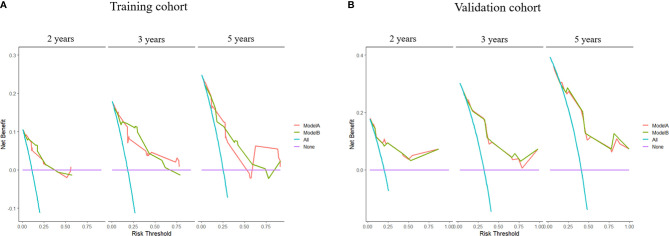
Decision curve analyses (DCA) of models **(A, B)** at 2, 3, and 5 years after surgery in the training cohort and 2, 3, and 5 years after surgery in the validation cohort.

**Figure 4:**
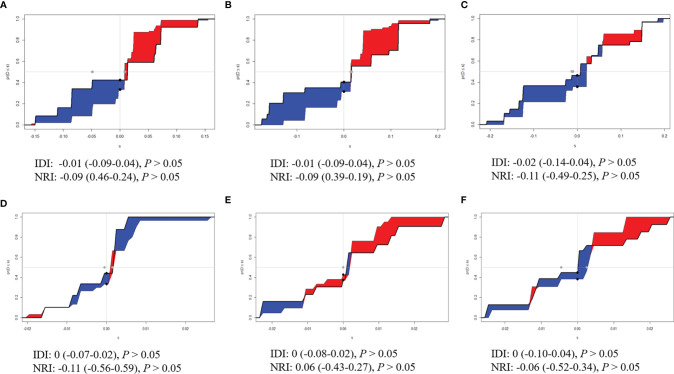
Integrated discrimination improvements (IDI) and Net Reclassification Index (NRI) of model B by comparing with model **(A)**. **(A)** Two years, **(B)** 3 years, and **(C)** 5 years after surgery in the training cohort. **(D)** Two years, **(E)** 3 years, and **(F)** 5 years after surgery in the validation cohort.

The time-dependent ROC curves of the two models showed that they both have good predictive performance (AUC >0.7) in the training cohort and validation cohort during the follow-up time (**Figure 2**). Overall, the predictive performance of model B was slightly better than model A in the training cohort but slightly weaker than model A in the first half of the follow-up time in the validation cohort. Comparison of the clinical usage of the two models in the training cohort and validation cohort evaluated by DCA, IDI, and NRI were as follows: the 2-, 3-, and 5-year DCA curves showed that the net benefit of ModelB could be better or worse than model A at different risk thresholds (**Figure 3**); as shown in **Figure 4**, the IDI approach indicated that the clinical utility of model B was similar to model A in both training cohort (2 years after surgery: IDI = −0.01, 95% CI = −0.09–0.04, *p* > 0.05; 3 years after surgery: IDI = −0.01, 95% CI = −0.09–0.04, *p* > 0.05; 5 years after surgery: IDI = −0.02, 95% CI = −0.14–0.04, *p* > 0.05) and validation cohort (2 years after surgery: IDI = 0, 95% CI = −0.07–0.02, *p* > 0.05; 3 years after surgery: IDI = 0, 95% CI = −0.08–0.02, *p* > 0.05; 5 years after surgery: IDI = 0, 95% CI = −0.10–0.04, *p* > 0.05); the NRI approach shown in **Figure 4** indicated that the clinical utility of model B was also similar to model A in both training cohort (2 years after surgery: NRI = −0.09, 95%CI = −0.46–0.24, *p* > 0.05; 3 years after surgery: NRI = −0.09, 95% CI = −0.39–0.19, *p* > 0.05; 5 years after surgery: NRI = −0.11, 95% CI = −0.49–0.25, *p* > 0.05) and validation cohort (2 years after surgery: NRI = −0.11, 95% CI = −0.56–0.59, *p* > 0.05; 3 years after surgery: NRI = 0.06, 95% CI = −0.43–0.27, *p* > 0.05; 5 years after surgery: NRI = −0.06, 95% CI = −0.52–0.34, *p* > 0.05).

The above results showed that mitotic level did not bring significant improvement in predictive ability. Thus, the mitotic level was excluded and the more simplified model (model B) was selected.

### Establishment and Verification of Nomogram

**Figure 5:**
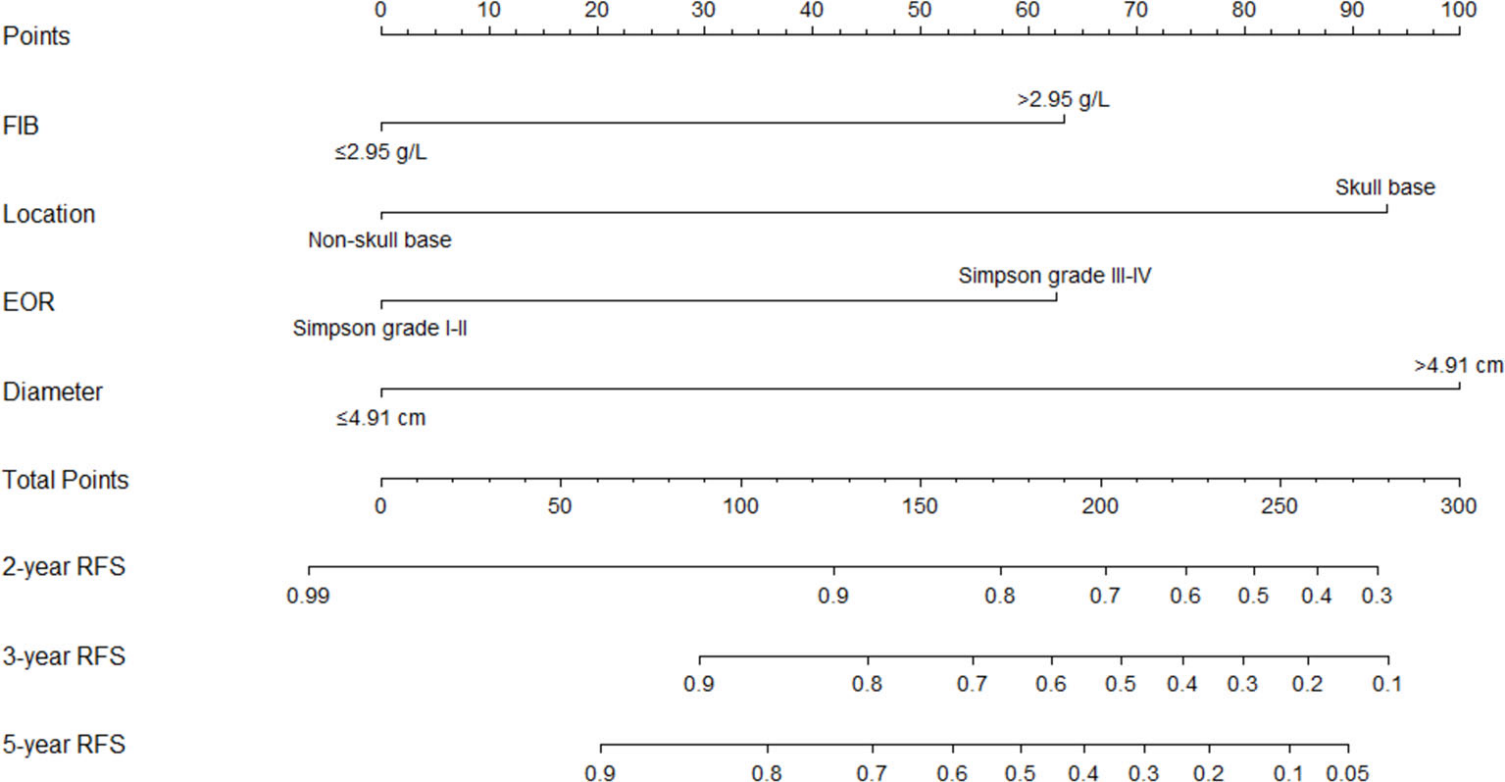
The nomogram for predicting 2-, 3-, and 5-year recurrence-free survival rate of a typical meningioma patients. FIB, fibrinogen; EOR, extent of resection; RFS, recurrence-free survival.

**Figure 6:**
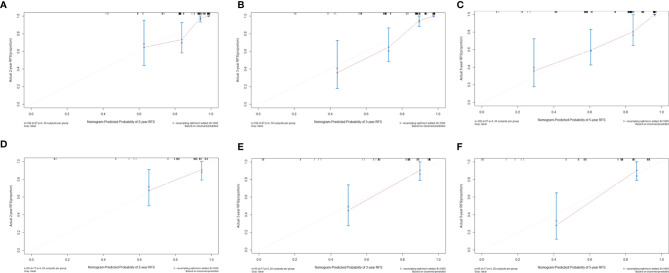
Calibration curves to predict **(A)** 2-year, **(B)** 3-year, and **(C)** 5-year recurrence-free survival rates in the training cohort and **(D)** 2-year, **(E)** 3-year, and **(F)** 5-year recurrence-free survival rates in the validation cohort.

FIB, tumor location, extent of resection, and tumor diameter were incorporated into the nomogram for recurrence prediction in the training cohort (**Figure 5**). The nomogram showed good discrimination ability (C-index, 0.822; 95% CI, 0.759–0.885). The calibration curves for the RFS rate at 2, 3, and 5 years showed good consistency between the predicted and observed probability (**Figures 6A–C**). In the validation cohort, the model also showed a good prediction with C-index = 0.817 (95% CI, 0.716–0.918). Good match was observed between the predicted and observed probability in this cohort (**Figures 6D–F**).

## Discussion

Currently, the treatment strategy for AM is surgical resection. Even with gross total resection, a considerable fraction of patients may recur years after surgery due to the aggressive progression and invasion (4, 5, 8, 10, 11). Patients who received the same treatment regimen may exhibit heterogeneity in tumor growth and recurrence. Another intrinsic challenge for the nuanced investigation of AM recurrence is its low incidence, resulting in a long-time span of the study to achieve sufficient sample size and follow-up duration. In addition, since the shifting of WHO diagnostic criteria over time, studies focused on AM have become more problematic. Precise and reliable model for recurrence prediction is helpful to guide clinicians in management and follow-up strategy of individual patients. Therefore, predicting recurrence of AM has been an urgent problem and a challenge in clinic.

Considering the interactions of the risk factors and integrating them into a nomogram model may be more practical and reliable for recurrence and prognosis prediction of many malignancies (15, 16). Our study divided the serum biomarkers and tumor diameter as binary variables based on their optimal cutoff value, which may be practical in guiding clinical decision-making. In addition, we divided the patients into a training cohort and a validation cohort based on the ratio of 7:3. The two cohorts were homogeneous and comparable based on the comparison of general characteristics. After multivariable Cox analysis in the training cohort, fibrinogen level, tumor location, extent of resection, tumor diameter, and mitotic level remained independently associated with AM recurrence. Instead of directly applying those factors into a predictive model, we utilized the LASSO regression analysis to avoid over-fitting or under-fitting. This method could analyze all variables at the same time and decrease the estimation variance. After the LASSO regression analysis, mitotic level lost its significance. As part of the diagnostic criteria, mitotic level has been confirmed to be associated with AM recurrence in previous studies (9, 17). To further investigate the impact of mitotic level in recurrence prediction of AM, we established two models according to the independent factors with or without mitotic level. After comparing their predictive performance (time-ROC) and clinical usage (DCA, IDI, NRI), we found that it was hard to determine the improvement of predictive ability brought by mitotic level in both training cohort and validation cohort. Thus, we integrated the other four independent risk factors into the final model and establish a nomogram. The nomogram showed an excellent discriminating ability in both training cohort (C-index: 0.822, 95% CI: 0.759–0.885) and validation cohort (C-index: 0.817, 95% CI: 0.716–0.918). The calibration curves in the both cohorts also showed good consistency between the predicted and observed RFS probability at 2, 3, and 5 years after surgery, which indicated the reliability and repeatability of the nomogram. The results prompted us that early treatment for tumor with small size may reduce the risk of recurrence. Considering those risk factors, surgical strategy may be adjusted to balance the risk of postoperative recurrence and surgical injury in those patients diagnosed as AM *via* intraoperative frozen section analysis. According to our results, we could predict the risk of postoperative recurrence *via* the nomogram based on the obtained risk factors. For those patients with high recurrence risk, shorter follow-up period may be requested to strive for early detection and early treatment.

Fibrinogen is a glycoprotein synthesized in hepatocytes which participates in blood coagulation and is also involved in cancer growth and metastasis (18, 19). Although the mechanism is not clear, the relationship between fibrinogen and tumor progression may be explained as follow: first, deposition of fibrinogen in the extracellular matrix could serve as a scaffold for growth factors and promote cell invasion, adhesion, and migration of tumor (20, 21); second, fibrinogen could induce epithelial-to-mesenchymal transition *via* rapamycin (mTOR)/protein kinase B (AKT) signaling pathway to promote malignant transformation (22); third, the deposition of platelet-fibrin could form physical barrier for tumor cells to prevent the kill contact from NK cell (23) and the platelet-fibrin (OGEN) axis has been confirmed to impede NK cell elimination of tumor cells to promote their metastatic potential (24); fourth, fibrinogen could also be synthesized and released by tumor cells, which in turn promotes tumor cell proliferation *via* the combined effects with growth factors (21, 25, 26). In our study, the preoperative serum fibrinogen level was an independent risk factor of AM recurrence and further included in a predictive model. Serum fibrinogen level in benign meningioma has been confirmed to be significantly lower than that in glioblastomas and metastases (27). Also, in a dog meningioma study, the fibrinogen staining scores in meningioma have been confirmed the gradual increasing trend from WHO grade I to WHO grade III (28). These may provide the basis for the predictive value of fibrinogen in AM recurrence.

Many studies have confirmed the close relationship between extent of resection and AM recurrence (10, 29, 30). In our study, we also found that AM patients with incomplete resection (Simpson grades III–IV) had a higher risk of recurrence. Complete surgical tumor removal is always the goal pursued by surgeons. However, we should acknowledge that tumors located in the skull base are less amenable to be completely resected as they are located adjacent to critical anatomic structures, including cranial nerves, intracranial vessels, and brainstem. Therefore, those patients with tumor located in skull base may inherently have higher risk of tumor residue and recurrence. However, the literature regarding the predictive value of tumor location in AM is rather scarce and controversial. Budohoski et al. reported that parafalcine/parasagittal location was an independent risk factor of early recurrence in AM (31). Klinger et al. found that tumors located in skull base had a trend towards decreased recurrence in AM (32). However, Da Broi et al. identified a tendency towards more retreatment in AM located in skull base (33). Our study revealed that tumor located in skull base was strongly associated with AM recurrence and excluded from confounding factors in the multivariable Cox analysis. Other than that, tumor size is also an independent risk factor in our study. Although such finding has not been confirmed in other studies, tumor diameter could reflect the growth rate of AM to a certain extent and was confirmed its predictive value in recurrence in other tumors (34, 35). Multiple oncogenic drivers, inhibitors, and regulators could affect tumor growth and survival *via* multiple pathways (36, 37). Therefore, tumor diameter may reflect the combined effects of multiple factors. Our study utilized the ROC analysis to determine the cutoff value of tumor diameter and found that tumor diameter >4.91 cm was independently associated with AM recurrence. The proliferative ability reflected by tumor diameter may explain its relationship with AM recurrence.

Although many studies affirmed the efficacy of PORT in AM recurrence patients (11, 38), there was also contradictory report exist in whether PORT could decrease the risk of AM recurrence (39). For example, Masalha et al. claimed no significant correlation between PORT and AM recurrence (39). There is no consensus guideline on recommendations for AM patients (https://www.nccn.org/professionals/physician_gls/f_guidelines.asp#site). Considering the potential toxicities of PORT, its execution should be considered with caution based on many risk factors of complications, such as age, tumor location and so on. In addition, execution of PORT may also be affected by patients’ wishes and their financial capability. In our study, we failed to determine the association between PORT and recurrence risk of AM patients. In the multivariable Cox hazard regression analysis, PORT lost its significance after being adjusted by other factors. Therefore, it was excluded from our final analysis. The European Association of Neuro Oncology guidelines recommend that PORT should be considered in patients with incomplete resection (40). In the 25 patients with Simpson grades III–IV of our study, the patients with PORT had a longer RFS than those without PORT (54.00 *vs*. 17.50 months, *p* = 0.034), revealing the potential therapeutic benefits of PORT in AM patients with incomplete resection. Also, the curative effect of PORT in AM patients should be investigated and validated in further research with a larger sample.

Our study was not free from limitations. First, the retrospective design of the study may suffer the interference and selection bias. Second, some laboratory or immunohistochemical factors were not included in our study due to the lack of examination in the early cases. Also, some molecular profiling and genotyping which have a high impact on predicting the recurrence were not available in our study. Third, the low incidence of the disease limits the collection of large amounts of sample in a short period. Therefore, the patients included in our study were operated both before and after the occurrence of 2016 WHO criterion. With the updated definition of WHO criteria for AM, the proportion of AM was increased. In addition, our study incorporated patients treated as recently as 2019, resulting in a dilution of aggressiveness and recurrence rate in this study cohort due to the combined effects of updated definition of criteria and advancements of medical technology. Fourth, our study did not use objective scale for edema evaluation. Accurate evaluation for edema *via* neuronavigation and objective scale are requested to reduce measurement errors in the further study. Fifth, the mechanism of the relationship between fibrinogen and AM recurrence was not investigated in our study. Further work is needed to address this point.

## Conclusion

Our study established a comprehensive model for the recurrence prediction in AM patients based on multiple factors, including fibrinogen level, tumor location, extent of resection, and tumor diameter. The nomogram could assist clinicians to predict the treatment effects and make individualized follow-up management in the heterogeneous patients. Further multicenter and prospective studies with lager sample size are required to verify the accurate application of nomogram.

## Data Availability Statement

The raw data supporting the conclusions of this article will be made available by the authors, without undue reservation.

## Ethics Statement

The studies involving human participants were reviewed and approved by the local ethics committee of the First Affiliated Hospital of Fujian Medical University and Fuzong Clinical Medical College of Fujian Medical University. The ethics committee waived the requirement of written informed consent for participation.

## Author Contributions

Y-XL, H-HY, and D-ZK performed and designed the study and wrote the manuscript. X-YC, J-YC, Y-XH, J-HX, W-WS, Y-C, C-YD, S-BW, and X-YW collected, interpreted, and analyzed the data. All authors contributed to the article and approved the submitted version

## Funding

This study was supported by grants from the National Natural Science Foundation of China (No. 81901395), Startup Fund for Scientific Research of Fujian Medical University (No. 2018QH1049), the project of improving the diagnosis and treatment ability of complicated diseases (No. PT-YNBZW2018), and Professor Academic Development Fund of Fujian Medical University (No. JS15014).

## Conflict of Interest

The authors declare that the research was conducted in the absence of any commercial or financial relationships that could be construed as a potential conflict of interest.

## Publisher’s Note

All claims expressed in this article are solely those of the authors and do not necessarily represent those of their affiliated organizations, or those of the publisher, the editors and the reviewers. Any product that may be evaluated in this article, or claim that may be made by its manufacturer, is not guaranteed or endorsed by the publisher.

## References

[1] Ostrom QT Gittleman H Fulop J Liu M Blanda R Kromer C. CBTRUS Statistical Report: Primary Brain and Central Nervous System Tumors Diagnosed in the United States in 2008-2012. Neuro Oncol (2015) 17 Suppl 4(1523-5866(1523-5866 (Electronic):iv1–iv62. doi: 10.1093/neuonc/nov189 26511214 PMC4623240

[2] Louis DN Perry A Reifenberger G von Deimling A Figarella-Branger D Cavenee WK. The 2016 World Health Organization Classification of Tumors of the Central Nervous System: A Summary. Acta Neuropathol (2016) 131(6):803–20. doi: 10.1007/s00401-016-1545-1 27157931

[3] Pasquier D Bijmolt S Veninga T Rezvoy N Villa S Krengli M. Atypical and Malignant Meningioma: Outcome and Prognostic Factors in 119 Irradiated Patients. A Multicenter, Retrospective Study of the Rare Cancer Network. Int J Radiat Oncol Biol Phys (2008) 71(5):1388–93. doi: 10.1016/j.ijrobp.2007.12.020 18294779

[4] Cao X Hao S Wu Z Wang L Jia G Zhang L. Treatment Response and Prognosis After Recurrence of Atypical Meningiomas. World Neurosurg (2015) 84(4):1014–9. doi: 10.1016/j.wneu.2015.05.032 26038336

[5] Nowak A Dziedzic T Krych P Czernicki T Kunert P Marchel A. Benign Versus Atypical Meningiomas: Risk Factors Predicting Recurrence. Neurol Neurochir Pol (2015) 49(1):1–10. doi: 10.1016/j.pjnns.2014.11.003 25666766

[6] Pisćević I Villa A Milićević M Ilić R Nikitović M Cavallo LM. The Influence of Adjuvant Radiotherapy in Atypical and Anaplastic Meningiomas: A Series of 88 Patients in a Single Institution. World Neurosurg (2015) 83(6):987–95. doi: 10.1016/j.wneu.2015.02.021 25769488

[7] Sofela AA McGavin L Whitfield PC Hanemann CO. Biomarkers for Differentiating Grade II Meningiomas From Grade I: A Systematic Review. Br J Neurosurg (2021) 1360-046X(Electronic):1–7. doi: 10.1080/02688697.2021.1940853 34148477

[8] Wang D Sun S Hua L Deng J Luan S Cheng H. Prognostic Model That Predicts Benefits of Adjuvant Radiotherapy in Patients With High Grade Meningioma. Front Oncol (2020) 10(2234-943X:568079(2234-943X (Print). doi: 10.3389/fonc.2020.568079 33240812 PMC7683714

[9] Domingo RA Tripathi S Vivas-Buitrago T Lu VM Chaichana KL Quinones-Hinojosa A. Mitotic Index and Progression-Free Survival in Atypical Meningiomas. World Neurosurg (2020) 142(1878-8769(1878-8769 (Electronic):191–6. doi: 10.1016/j.wneu.2020.06.189 32615290

[10] Keric N Kalasauskas D Freyschlag CF Gempt J Misch M Poplawski A. Impact of Postoperative Radiotherapy on Recurrence of Primary Intracranial Atypical Meningiomas. J Neurooncol (2020) 146(2):347–55. doi: 10.1007/s11060-019-03382-x 31900826

[11] Phonwijit L Khawprapa C Sitthinamsuwan B. Progression-Free Survival and Factors Associated With Postoperative Recurrence in 126 Patients With Atypical Intracranial Meningioma. World Neurosurg (2017) 107(1878-8769(1878-8769 (Electronic):698–705. doi: 10.1016/j.wneu.2017.08.057 28838877

[12] Liu L Zheng Z Li J Li Y Ni J. Supraclavicular Recurrence in Completely Resected (Y)Pn2 Non-Small Cell Lung Cancer: Implications for Postoperative Radiotherapy. Front Oncol (2020) 10(2234-943X:1414(2234-943X (Print). doi: 10.3389/fonc.2020.01414 32850456 PMC7431951

[13] Shi X Xu L Ma B Wang S. Development and Validation of a Nomogram to Predict the Prognosis of Patients With Gastric Cardia Cancer. Sci Rep (2020) 10(1):14143. doi: 10.1038/s41598-020-71146-z 32839498 PMC7445298

[14] Royston P Moons KG Altman DG Vergouwe Y. Prognosis and Prognostic Research: Developing a Prognostic Model. BMJ (2009) 338(1756-1833(1756-1833 (Electronic):b604. doi: 10.1136/bmj.b604 19336487

[15] Han DS Suh YS Kong SH Lee HJ Choi Y Aikou S. Nomogram Predicting Long-Term Survival After D2 Gastrectomy for Gastric Cancer. J Clin Oncol (2012) 30(31):3834–40. doi: 10.1200/JCO.2012.41.8343 23008291

[16] Liang W Zhang L Jiang G Wang Q Liu L Liu D. Development and Validation of a Nomogram for Predicting Survival in Patients With Resected Non-Small-Cell Lung Cancer. J Clin Oncol (2015) 33(8):861–9. doi: 10.1200/JCO.2014.56.6661 25624438

[17] Barresi VA-O Lionti S Caliri S Caffo M. Histopathological Features to Define Atypical Meningioma: What Does Really Matter for Prognosis? Brain Tumor Pathol (2018) 35(3):168–80. doi: 10.1007/s10014-018-0318-z 29671247

[18] Takeuchi H Ikeuchi S Kitagawa Y Shimada A Oishi T Isobe Y. Pretreatment Plasma Fibrinogen Level Correlates With Tumor Progression and Metastasis in Patients With Squamous Cell Carcinoma of the Esophagus. J Gastroenterol Hepatol (2007) 22(12):2222–7. doi: 10.1111/j.1440-1746.2006.04736.x 18031385

[19] Wu Y Song Z Sun K Rong S Gao P Wang F. A Novel Scoring System Based on Peripheral Blood Test in Predicting Grade and Prognosis of Patients With Glioma. Onco Targets Ther (2019) 12(1178-6930(1178-6930 (Print):11413–23. doi: 10.2147/OTT.S236598 PMC6935303 31920331

[20] Simpson-Haidaris PJ Rybarczyk B. Tumors and Fibrinogen. The Role of Fibrinogen as an Extracellular Matrix Protein. Ann N Y Acad Sci (2001) 936(0077-8923(0077-8923 (Print):406–25. 11460495

[21] Sahni A Simpson-Haidaris PJ Sahni SK Vaday GG Francis CW. Fibrinogen Synthesized by Cancer Cells Augments the Proliferative Effect of Fibroblast Growth Factor-2 (FGF-2). J Thromb Haemost (2008) 6(1):176–83. doi: 10.1111/j.1538-7836.2007.02808.x 17949478

[22] Zhang F Wang Y Sun P Wang ZQ Wang DS Zhang DS. Fibrinogen Promotes Malignant Biological Tumor Behavior Involving Epithelial-Mesenchymal Transition via the P-AKT/p-mTOR Pathway in Esophageal Squamous Cell Carcinoma. J Cancer Res Clin Oncol (2017) 143(12):2413–24. doi: 10.1007/s00432-017-2493-4 PMC11819069 28801734

[23] Nieswandt B Hafner M Echtenacher B Männel DN. Lysis of Tumor Cells by Natural Killer Cells in Mice Is Impeded by Platelets. Cancer Res (1999) 59(6):1295–300. 10096562

[24] Palumbo JS Talmage KE Massari JV La Jeunesse CM Flick MJ Kombrinck KW. Platelets and Fibrin(Ogen) Increase Metastatic Potential by Impeding Natural Killer Cell-Mediated Elimination of Tumor Cells. Blood (2005) 105(1):178–85. doi: 10.1182/blood-2004-06-2272 15367435

[25] Yamaguchi T Yamamoto Y Yokota S Nakagawa M Ito M Ogura T. Involvement of Interleukin-6 in the Elevation of Plasma Fibrinogen Levels in Lung Cancer Patients. Jpn J Clin Oncol (1998) 28(12):740–4. doi: 10.1093/jjco/28.12.740 9879291

[26] Sahni A Francis CW. Vascular Endothelial Growth Factor Binds to Fibrinogen and Fibrin and Stimulates Endothelial Cell Proliferation. Blood (2000) 96(12):3772–8. 11090059

[27] Stover JF Hopf NJ Perneczky A Kempski OS. Unspecific Metabolic Blood Parameters as Used in Clinical Routine may Differentiate Malignant From Benign Cerebral Tumors. Cancer Lett (1995) 95(1-2):147–52. doi: 10.1016/0304-3835(95)03880-6 7656223

[28] Font C de la Fuente C Pumarola M Blasco E Fernandez F Viu J. Canine Intracranial Meningiomas: Immunohistochemical Evaluation of Tissue Factor, Fibrin/Fibrinogen and D-Dimers. Vet J (2015) 206(3):426–8. doi: 10.1016/j.tvjl.2015.07.008 26526524

[29] Dobran M Marini A Splavski B Rotim K Liverotti V Nasi D. Surgical Treatment and Predictive Factors for Atypical Meningiomas: A Multicentric Experience. World Neurosurg (2020) 144(1878-8769(1878-8769 (Electronic):e1–8. doi: 10.1016/j.wneu.2020.03.201 32311549

[30] Soni P Davison MA Shao J Momin A Lopez D Angelov L. Extent of Resection and Survival Outcomes in World Health Organization Grade II Meningiomas. J Neurooncol (2021) 151(2):173–9. doi: 10.1007/s11060-020-03632-3 33205354

[31] Budohoski KP Clerkin J Millward CP O'Halloran PJ Waqar M Looby S. Predictors of Early Progression of Surgically Treated Atypical Meningiomas. Acta Neurochir (Wien) (2018) 160(9):1813–22. doi: 10.1007/s00701-018-3593-x PMC6105233 29961125

[32] Klinger DR Flores BC Lewis JJ Hatanpaa K Choe K Mickey B. Atypical Meningiomas: Recurrence, Reoperation, and Radiotherapy. World Neurosurg (2015) 84(3):839–45. doi: 10.1016/j.wneu.2015.04.033 25916182

[33] Da Broi M Borrelli PA-OX Meling TA-O. Predictors of Survival in Atypical Meningiomas. Cancers (Basel) (2021) 13(8):1970. doi: 10.3390/cancers13081970 33919475 PMC8074901

[34] Ma E Li J Xing H Li R Shen C Zhang Q. Development of a Predictive Nomogram for Early Recurrence of Hepatocellular Carcinoma in Patients Undergoing Liver Transplantation. Ann Transl Med (2021) 9(6):468. doi: 10.21037/atm-21-334 33850865 PMC8039665

[35] Zhang H Ma Y Wang H Xu L Yu Y. MMP-2 Expression and Correlation With Pathology and MRI of Glioma. Oncol Lett (2019) 17(2):1826–32. doi: 10.3892/ol.2018.9806 PMC6341586 30675244

[36] He JH Han ZP Luo JG Jiang JW Zhou JB Chen WM. Hsa_Circ_0007843 Acts as a mIR-518c-5p Sponge to Regulate the Migration and Invasion of Colon Cancer SW480 Cells. Front Genet (2020) 11(1664-8021:9(1664-8021 (Print). doi: 10.3389/fgene.2020.00009 32158464 PMC7052121

[37] Parikh AA-O Shin J Faquin W Lin DT Tirosh I Sunwoo JB. Malignant Cell-Specific CXCL14 Promotes Tumor Lymphocyte Infiltration in Oral Cavity Squamous Cell Carcinoma. LID - 10.1136/Jitc-2020-001048 [Doi] LID - E001048. J Immunother Cancer (2020) 8(2):e001048. doi: 10.1136/jitc-2020-001048 32958684 PMC7507891

[38] Hemmati SM Ghadjar P Grun A Badakhshi H Zschaeck S Senger C. Adjuvant Radiotherapy Improves Progression-Free Survival in Intracranial Atypical Meningioma. Radiat Oncol (2019) 14(1):160. doi: 10.1186/s13014-019-1368-z 31477146 PMC6719347

[39] Masalha WA-O Heiland DH Franco P Delev D Haaker JG Schnell O. Atypical Meningioma: Progression-Free Survival in 161 Cases Treated at Our Institution With Surgery Versus Surgery and Radiotherapy. J Neurooncol (2018) 136(1):147–54. doi: 10.1007/s11060-017-2634-2 29081038

[40] Goldbrunner R Minniti G Preusser M Jenkinson MD Sallabanda K Houdart E. EANO Guidelines for the Diagnosis and Treatment of Meningiomas. Lancet Oncol (2016) 17(9):e383–91. doi: 10.1016/S1470-2045(16)30321-7 27599143

